# Development and Clinical Validation of Multiple Cross Displacement Amplification Combined With Nanoparticles-Based Biosensor for Detection of *Mycobacterium tuberculosis*: Preliminary Results

**DOI:** 10.3389/fmicb.2019.02135

**Published:** 2019-09-13

**Authors:** Wei-Wei Jiao, Yi Wang, Gui-Rong Wang, Ya-Cui Wang, Jing Xiao, Lin Sun, Jie-Qiong Li, Shu-An Wen, Ting-Ting Zhang, Qi Ma, Hai-Rong Huang, A-Dong Shen

**Affiliations:** ^1^Key Laboratory of Major Diseases in Children, Ministry of Education, National Key Discipline of Pediatrics (Capital Medical University), National Clinical Research Center for Respiratory Diseases, Beijing Key Laboratory of Pediatric Respiratory Infection Disease, Beijing Pediatric Research Institute, Beijing Children’s Hospital, Capital Medical University, National Center for Children’s Health, Beijing, China; ^2^National Tuberculosis Clinical Laboratory, Beijing Key Laboratory for Drug Resistance Tuberculosis Research, Beijing Tuberculosis and Thoracic Tumor Research Institute, Beijing Chest Hospital, Capital Medical University, Beijing, China

**Keywords:** *Mycobacterium tuberculosis*, multiple cross displacement amplification, lateral flow biosensor, MCDA-LFB, limit of detection

## Abstract

Tuberculosis is still a major threat to global public health. Here, a novel diagnosis assay, termed as multiple cross displacement amplification combined with nanoparticle-based lateral flow biosensor (MCDA-LFB), was developed to simultaneously detect IS*6110* and IS*1081* of *Mycobacterium tuberculosis* (MTB) in DNA extracted from reference strain H37Rv and clinical samples. The amplification can be finished within 30 min at a fixed temperature (67°C), thus the whole procedure, including rapid template preparation (15 min), isothermal reaction (30 min) and result reporting (2 min), can be completed within 50 min. The limit of detection of multiplex MCDA assay was 10 fg per reaction. By using the multiplex MCDA protocol, cross-reaction with non-mycobacteria and non-tuberculous mycobacteria (NTM) strains was not observed. Among clinically diagnosed TB patients, the sensitivity of liquid culture, Xpert MTB/RIF and multiplex MCDA assay was 42.0% (50/119), 49.6% (59/119), and 88.2% (105/119), respectively. Among culture positive samples, the sensitivity of Xpert MTB/RIF and multiplex MCDA assay was 86.0% (43/50) and 98.0% (49/50), respectively. Among culture negative samples, the sensitivity of Xpert MTB/RIF and multiplex MCDA assay was 23.2% (16/69) and 81.2% (56/69), respectively. The specificity was 100% (60/60) for Xpert MTB/RIF and 98.3% (59/60) for multiplex MCDA. Therefore, the multiplex MCDA assay for MTB detection is rapid, sensitive and easy to use and may be a promising test for early diagnosis of TB.

## Introduction

Tuberculosis, caused by *Mycobacterium tuberculosis* (MTB), is still a major threat to global public health, with an estimated 10 million new cases and 1.3 million deaths in 2017 (World-Health-Organizaion, 2018). Therefore, rapid and accurate detection of the causative agent is a key factor highly associated with prognosis and treatment cost.

Conventional MTB detection methods, including sputum smear microscopy and mycobacterial culture, remain the major tools in the TB endemic countries. The former lacks sensitivity. The latter has been used as the gold standard of TB diagnosis for many decades but it is a time-consuming, laborious and technically demanding technique. In particular, bacterial culture cannot meet the requirement for rapid diagnosis in clinical settings.

In recent years, many molecular techniques have been devised for rapidly detecting MTB directly from clinical samples. Xpert MTB/RIF and loop-mediated isothermal amplification (LAMP) are recommended by the WHO as rapid molecular methods for MTB detection (World-Health-Organizaion, 2011, 2016). Xpert MTB/RIF provides a convenient and sensitive way to detect MTB (Boehme et al., 2010). However, the high device and consumable costs impede its usage in peripheral laboratories. TB-LAMP relies on a relatively simple device, and the results are based on turbidity visualized with unaided eyes or under ultraviolet light (Ou et al., 2014; Bojang et al., 2016). However, a visualization of the TB-LAMP products by color change with the naked eye is somewhat subjective; therefore, ambiguous results may occur when the concentration of MTB templates is very low.

To address the shortcomings posed by Xpert MTB/RIF and TB-LAMP, we developed a novel molecular assay based on the before-mentioned multiple cross displacement amplification (MCDA) (Wang et al., 2015). MCDA assay achieves rapid and effective amplification of nucleic acid sequences under isothermal conditions within ∼20–40 min (Wang et al., 2018a). MCDA displays extremely high specificity for target sequence amplification, as it comprises ten primers designed for each target, including two displacement primers (F1 and F2), two cross primers (CP1 and CP2), and six amplification primers (C1, D1, R1, C2, D2, and R2). More recently, nanoparticle-based lateral flow biosensors (LFBs) have been devised and applied for simply, rapidly, visually, and objectively indicating the MCDA results (Wang et al., 2018b, c).

In this study, we firstly report a MCDA combined with the LFB (MCDA-LFB) method for simple, visible and reliable detection of MTB using two target genes (IS*6110* and IS*1081*) and validate its potential clinical application using clinical samples from patients.

## Methods

### Experiment Design

**FIGURE 1:**
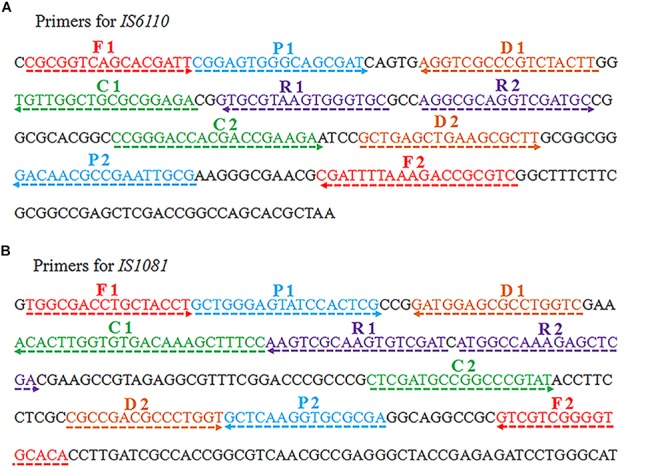
Schematic view of sequences and location of MCDA primers. The nucleotide sequences of the sense strand of IS*6110* **(A)** and IS*1081* **(B)** are shown. The locations of primers are marked with different colors. Right and left arrows indicate the sense and complementary sequences.

Two sets of MCDA primers, including the IS*6110*-MCDA primer set and IS*1081*-MCDA primer set, were designed based on the MTB conserved sequences, IS*6110* and IS*1081*, respectively. The details of primers used in this study were shown in Figure 1 and Table 1. All the primers were synthesized by TianyiHuiyuan Biotechnology Co. Ltd. (Beijing, China). The glass bead-based kits (CapitalBio Co.) were used to extract DNA from bacteria and clinical samples. Isothermal amplification kits and visual detection reagent (VDR) were obtained from BeiJing-HaiTaiZhengYuan Technology Co., Ltd. (Beijing, China).

**TABLE 1:** The sequences of primers used in this study.

**Assay**	**Primers**	**Sequence (5′–3′)**	**Target gene**
MTB-MCDA	6110-F1	CGCGGTCAGCACGATT	IS*6110*
	6110-F2	GACGCGGTCTTTAAAATCG	IS*6110*
	6110-CP1	TCTCCGCGCAGCCAACACGGAG TGGGCAGCGAT	IS*6110*
	6110-CP2	CCGGGACCACGACCGAAGACGCA ATTCGGCGTTGTC	IS*6110*
	6110-C2	CCGGGACCACGACCGAAGA	IS*6110*
	6110-D2	GCTGAGCTGAAGCGCTT	IS*6110*
	6110-R1	GCACCCACTTACGCAC	IS*6110*
	6110-R2	AGGCGCAGGTCGATGC	IS*6110*
	1081-F1	TGGCGACCTGCTACCT	IS*1081*
	1081-F2	TGTGCACCCCGACGAC	IS*1081*
	1081-CP1	GGAAAGCTTTGTCACACCAAGTG TGCTGGGAGTATCCACTCG	IS*1081*
	1081-CP2	CTCGATGCCGGCCCGTATTCG CGCACCTTGAGC	IS*1081*
	1081-C2	CTCGATGCCGGCCCGTAT	IS*1081*
	1081-D2	CGCCGACGCCCTGGT	IS*1081*
	1081-R1	ATCGACACTTGCGACTT	IS*1081*
	1081-R2	ATGGCCAAAGAGCTCGA	IS*1081*
Primers labeled	6110-C1^∗^	5′-FITC-TCTCCGCGCAGCCAACA-3′	IS*6110*
	6110-D1#	5′-biotin-AAGTAGACGGGCGACCT-3′	IS*6110*
	1081-C1^∗^	5′-FITC-GGAAAGCTTTGTCACACCAAGTGT-3′	IS*1081*
	1081-D1#	5′-biotin-GACCAGGCGCTCCATC-3′	IS*1081*

*FITC, fluorescein isothiocyanate.*

### MCDA Reactions

The singlex MCDA reaction system for IS*6110* or IS*1081* was performed in the 25-μl mixture containing 12.5 μl of 2X supplied buffer, 0.4 μM each of F1 and F2; 0.8 μM each of C1^∗^, C2, R1, R2, D1^#^ and D2; 1.6 μM each of CP1 and CP2; 1 μl of VDR; 1 μl (8 U) of *Bst*2.0 DNA polymerase; and DNA template (1 μl for cultured strains, 5 μl for clinical samples).

The multiplex MCDA was also performed in a 25-μl mixture containing 12.5 μl 2 X of the supplied buffer; 0.2 μM each of IS*6110*-F1, IS*6110*-F2, IS*1081*-F1 and IS*1081*-F2; 0.4 μM each of IS*6110*-C1^∗^, IS*6110*-C2, IS*6110*-R1, IS*6110*-R2, IS*6110*-D1^#^, IS*6110*-D2, IS*1081*-C1^∗^, IS*1081*-C2, IS*1081*-R1, IS*1081*-R2, IS*1081*-D1^#^ and IS*1081*-D2; 0.8 μM each of IS*6110*-CP1, IS*6110*-CP2, IS*1081*-CP1 and IS*1081*-CP2; 1 μl of VDR, 1 μl (8 U) of *Bst*2.0 DNA polymerase; and DNA template (1 μl for isolated strains, 5 μl for clinical samples).

The VDR, real-time turbidity (LA-320C) and LFB platform were used to demonstrate and confirm the MCDA products. The LFB platform was prepared according to the previous studies (Wang et al., 2017). In brief, Dye (Crimson red) streptavidin-coated polymer nanoparticles (SA-DNPs, Fishers, IN, United States) were gathered in the conjugate pad, and anti-FITC and biotin-BSA were affixed at the test line (TL) and control line (CL), respectively. The MCDA products (0.5 μl) and running buffer (70 μl) were added into the immersion pad. Through the capillary flow, the MCDA products and SA-DNPs were transferred to TL and CL. As a result, FITC/MCDA/SA-DNPs complexes and non-complexed SA-DNPs were indicated by crimson red lines at the TL and CL, respectively. The colorimetric bands were easily visible within 2 min.

In this report, the amplification temperatures of the two sets of MCDA primers (target for IS*6110* and IS*1081*) were optimized from 61°C to 68°C at 1°C intervals. The DNA template of H37Rv (1 ng) was used as positive control, and *Mycobacterium abscessus* DNA (1 ng) and distilled water (DW) were used as negative control and blank control, respectively. The reactions were analyzed by real-time turbidity detection.

### Sensitivity of MCDA Assays

The DNA template of the reference strain H37Rv was quantified using Nanodrop ND-1000 at A260/280 and then serially diluted to 10 ng, 1 ng, 100 pg, 10 pg, 1 pg, 100 fg, 10 fg, and 1 fg per microliter. A volume of 1 μl of each dilution was added into the MCDA reaction. The limits of detection (LOD) of singlex (IS*6110*-MCDA and IS*1081*-MCDA) and multiplex MCDA reactions were determined as the last positive dilution.

Then, the duration of reaction time for multiplex MCDA was optimized. Four different amplification times, including 10 min, 20 min, 30 min, and 40 min, were compared under the optimal reaction conditions, and the results were confirmed using LFB.

### Specificity of Multiplex MCDA Assay

To verify the specificity of multiplex MCDA assay, a total of 52 genomic DNA samples (at least 1 ng/μl) from different bacterial strains were examined, including 1 MTB reference strain H37Rv, 1 BCG strain, 20 MTB strains isolated from clinical patients, 21 non-mycobacteria strains and 9 non-tuberculous mycobacteria (NTM) strains (Table 2). The multiplex MCDA results were confirmed using LFB.

**TABLE 2:** Genomic DNA used to determine the specificity of MCDA.

**Bacteria**	**Strain no. (source of strains)^a^**	**No. of strains**	**MCDA result^b^**
*M. tuberculosis*	H37Rv	1	P
	BCG	1	P
	Isolated strains (BCH)	20	P
*Klebsiella pneumoniae*	Isolated strains (BCH)	1	N
*Streptococcus pneumonia*	Isolated strains (BCH)	1	N
*Pseudomonas aeruginosa*	Isolated strains (BCH)	1	N
*Listeria monocytogenes*	Isolated strains (BCH)	1	N
*Staphylococcus epidermidis*	Isolated strains (BCH)	1	N
*Staphylococcus aureus*	Isolated strains (BCH)	1	N
*Bacillus cereus*	Isolated strains (BCH)	1	N
*Enterobacter sakazakii*	Isolated strains (BCH)	1	N
*Campylobacter jejuni*	Isolated strains (BCH)	1	N
*Escherichia coli*	Isolated strains (BCH)	1	N
*Aeromonas hydrophila*	Isolated strains (BCH)	1	N
*Pseudomonas Shigella*	Isolated strains (BCH)	1	N
*Salmonella*	Isolated strains (BCH)	1	N
*Shigella flexneri*	Isolated strains (BCH)	1	N
*Shigella boydii*	Isolated strains (BCH)	1	N
*Shigella dysenteriae*	Isolated strains (BCH)	1	N
*Shigella sonnei*	Isolated strains (BCH)	1	N
*Vibrio parahaemolyticus*	Isolated strains (BCH)	1	N
*Vibrio vulnificus*	Isolated strains (BCH)	1	N
*Enterococcus faecium*	Isolated strains (BCH)	1	N
*Enterococcus faecalis*	Isolated strains (BCH)	1	N
*M. fortuitum*	Isolated strain (BCH-NTCL)	1	N
*M. abscessus*	Isolated strain (BCH-NTCL)	1	N
*M. avium*	Isolated strain (BCH-NTCL)	1	N
*M. intracellulare*	Isolated strain (BCH-NTCL)	1	N
*M. malmoense*	Isolated strain (BCH-NTCL)	1	N
*M. kansasii*	Isolated strain (BCH-NTCL)	1	N
*M. gordonae*	Isolated strain (BCH-NTCL)	1	N
*M. chelonei*	Isolated strain (BCH-NTCL)	1	N
*M. flavescens*	Isolated strain (BCH-NTCL)	1	N

*^*a*^BCH, Beijing Children’s Hospital; BCH-NTCL, National Tuberculosis Clinical Laboratory, Beijing Chest Hospital. ^*b*^P, positive; N, negative. Only genomic DNA templates from MTBC could be detected by MCDA assay, indicating the extremely high specificity of the method.*

### Validation of the Feasibility of Multiplex MCDA Assay Using Clinical Samples

The optimized multiplex MCDA assay was directly evaluated using clinical samples in parallel with Xpert MTB/RIF and/or culture. All the participants included in this study signed informed consent (the guardian signed it on his/her behalf if the child was younger than 15 years old). This study was reviewed and approved by the Ethics committee of Beijing Children’s Hospital, Capital Medical University (No. 2015-45) and Beijing Chest Hospital, Capital Medical University (No. 2014-36-1).

Two groups of patients were enrolled. The first group included adult patients with pulmonary tuberculosis (PTB) from January 2, 2019 to January 17, 2019. The second group included pediatric patients with excluded TB (diagnosed as pneumonia with confirmed etiological evidence of infection with virus, mycoplasma or bacteria) from January 1, 2019 to February 22, 2019. All the patients were categorized into three groups according to the Chinese Pulmonary Diagnosis Criteria WS288-2017 (National-Health-and-Family-Planning-commission-of-the-People’s-Republic-of-China, 2017): (i) definite case: a biological specimen positive by smear microscopy, or culture, or molecular methods, or histopathological change; (ii) clinically diagnosed case: radiographic evidence consistent with TB, and at least one of the following: symptoms of TB, positive tuberculin skin test or interferon-γ release assay results, histopathological or bronchoscopic examination consistent with TB; (iii) non-TB: alternative diagnosis established and clinical resolution made without anti-TB treatment.

For the enrolled patients, about 3 ml of clinical samples were collected. Liquid culture using Bactec 960 (BD Microbiology Systems, Sparks, MD, United States) and Xpert MTB/RIF assay (Cepheid, Sunnyvale, CA, United States) was performed according to the manufacturers’ instructions. The remaining samples (1 ml) were digested with equal volume of 4% NaOH. The genomic DNA was extracted using glass bead-based kits (CapitalBio Co.). A total of 100 μl of DNA solution was obtained and 5 μl was used for the multiplex MCDA assay. Reference strain H37Rv and DW were used as positive and negative controls, respectively.

## Results

### Amplification Temperature Optimization

**FIGURE 2:**
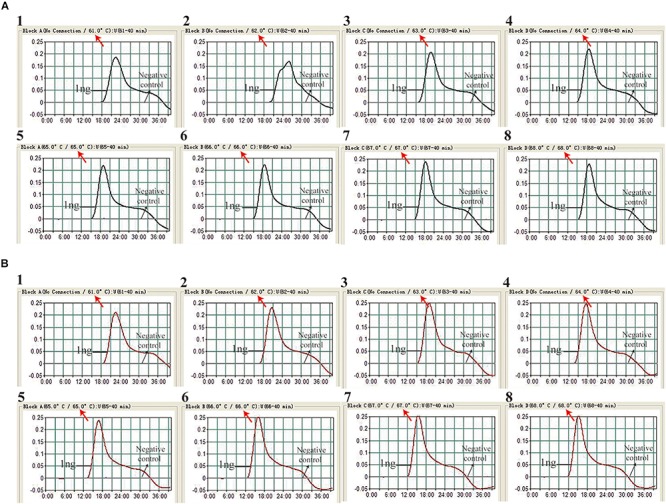
Optimization of amplification temperature for MCDA primer sets. The MCDA assays for MTB detection were monitored by real-time measurement of turbidity. The threshold value was 0.1, thus turbidity of >0.1 was regarded to be positive. Eight kinetic graphs (1–8) were obtained at different temperatures (61 –68°C at 1°C intervals), with 1 ng H37Rv template per reaction for IS*6110* primer set **(A)** and IS*1081* primer set **(B)**, respectively.

The reaction temperature is an important factor for the MCDA technique, which affects the amplification efficiency and reaction speed of target assay. According to our result (Figure 2), the optimum reaction temperature ranged from 64 to 68°C for the IS*6110*-MCDA assay, and from 66 to 68°C for the IS*1081*-MCDA assay. The faster amplifications were observed at an assay temperature of 67°C for both assays, thus this reaction temperature was used for performing the rest of IS*6110*-MCDA, IS*1081*-MCDA and multiplex MCDA reactions described in this report.

### Sensitivity of MCDA Assays

**FIGURE 3:**
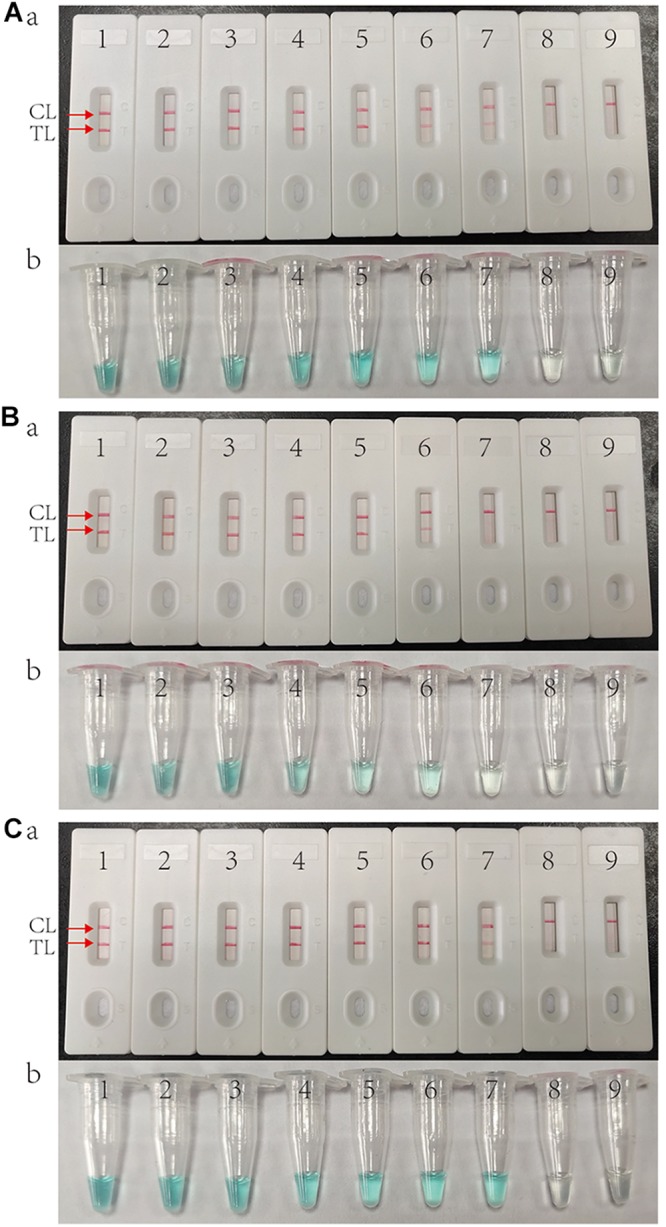
Sensitivity of MCDA assay using serially diluted genomic template of H37Rv. **(A)** IS*6110* MCDA assay; **(B)** IS*1081* MCDA assay; **(C)** Multiplex MCDA assay for both IS*6110* and IS*1081*. Biosensors **(Aa, Ba, Ca)**/tubes **(Ab, Bb, Cb)** 1–9 represented the DNA templates of H37Rv 10 ng, 1 ng, 100 pg, 10 pg, 1 pg, 100 fg, 10 fg, 1 fg per reaction and blank control (DW).

As shown in Figure 3, the LOD of IS*6110*-MCDA, IS*1081*-MCDA and multiplex MCDA assays is 10 fg, 100 fg, and 10 fg per reaction, respectively. By LFB, two crimson lines (TL and CL) simultaneously appear on the biosensor for positive MCDA reactions, and only a crimson band (CL) appears on the biosensor for negative reactions (Figures 3A–C; upper row). By VDR, light green in the positive reaction tube was observed, while negative reaction was colorless (Figures 3A–C; bottom row). In particular, the analysis sensitivity obtained from LFB detection is in conformity with VDR analysis (Figure 3).

### Optimized Reaction Time for Multiplex MCDA Assay

**FIGURE 4:**
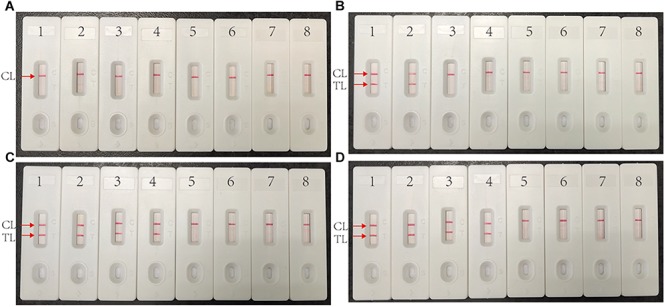
Optimized reaction time for multiplex MCDA assay. Four different duration times (**A**, 10 min; **B**, 20 min; **C**, 30 min; **D**, 40 min) were determined at optimal amplification temperature (67°C). Biosensors 1, 2, 3, 4, 5, and 6 represent genomic DNA template of H37Rv 10 pg, 1 pg, 100 fg, 10 fg, 1 fg, 100 ag. Biosensors 7 and 8 represent negative control (genomic DNA from *M. abscessus* 1 ng) and blank control (DW).

The target template at the LOD level (10 fg) can be detected when multiplex MCDA lasted 30 min (Figure 4C). Hence, 30 min was determined as the optimal amplification time for multiplex MCDA assay. As a result, the whole procedure, including rapid DNA extraction (15 min), MCDA reaction (30 min), and result indicating (2 min), could be completed within 50 min.

### Specificity of Multiplex MCDA Assay

**FIGURE 5:**
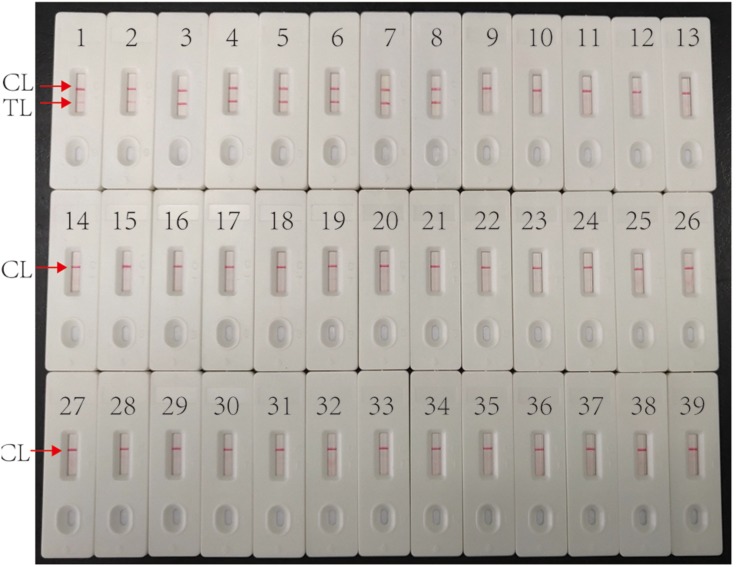
Specificity of multiplex MCDA assay using DNA templates from different bacteria. Biosensor 1 represents reference strain H37Rv. Biosensors 2–7 represent cultured MTB strains from clinical patients. Biosensor 8 represents BCG strain. Biosensors 9–29 represent 21 different non-mycobacteria bacteria (details shown in Table 2). Biosensors 30–38 represent nine different NTM strains (details shown in Table 2). Biosensor 39 represents blank control (DW).

Multiplex MCDA assay specifically detected all MTBC strains (such as H37Rv, BCG and MTB clinical strains), while non-MTBC bacterial pathogens were not detected. By biosensor, two crimson red lines (TL and CL) simultaneously appeared at detection zones of biosensor, suggesting the positive results for MTBC pathogens (Figure 5, biosensor 1-8). Only one CL appeared at the detection regions of biosensor, reporting the negative results for non-MTBC strains and blank control (DW) (Figure 5, biosensor 9-39). Our results confirm that the multiplex MCDA assay established here has a high specificity (100%) and is able to successfully differentiate MTBC from other bacteria and NTM strains.

### Application of Multiplex MCDA Assay in Clinical Samples

According to WS288-2017, total 119 PTB patients (50 were culture positive) and 60 non-TB patients were enrolled in this study. In the TB group, males accounted for 72.3% (86/119), and the mean age was 46.57 years old. The specimen type included 93 sputum, 18 bronchial lavage fluid, and 8 pleural fluid samples. In the non-TB group, 33 (55%) were males and the average age was 5.15 years old. The samples included 57 sputum and 3 pleural fluid samples. These non-TB patients were diagnosed as having pneumonia with confirmed pathogen (19 with bacterial infection, 2 with viral infection, 27 with *Mycoplasma pneumonia* infection, and 12 with mixed infection).

Among all the enrolled PTB patients, the sensitivity of liquid culture, Xpert MTB/RIF and multiplex MCDA assay was 42.0% (50/119), 49.6% (59/119) and 88.2% (105/119), respectively (Table 3). TB patients were further subdivided into culture-positive group and culture-negative group. Among culture-positive samples, the sensitivity of Xpert MTB/RIF and multiplex MCDA assay was 86.0% (43/50) and 98.0% (49/50), respectively. Among those samples with negative culture results, the sensitivity of Xpert MTB/RIF and multiplex MCDA assay was 23.2% (16/69) and 81.2% (56/69), respectively, indicating that multiplex MCDA assay can further improve the detection rate of culture-negative specimens. The specificity was 100% (60/60) for Xpert MTB/RIF and 98.3% (59/60) for multiplex MDCA when testing the samples from non-TB patients.

**TABLE 3:** Comparison of different methods for MTB detection in clinical TB patients.

	**Sensitivity, % (n^a^/N)**		**Specificity, % (n/N)**
	**Culture positive (*N* = 50)**	**Culture negative (*N* = 69)**	
Xpert MTB/RIF	86.0% (43/50)	23.2% (16/69)	100% (60/60)
MTB-MCDA	98.0% (49/50)	81.2% (56/69)	98.3% (59/60)

*^*a*^*n* number of samples with positive results using a specific method.*

## Discussion

In this study, we firstly established a multiplex MCDA-LFB method for simple, rapid, sensitive and reliable detection of MTB. A simple instrument (such as a heater or water-bath) is sufficient for maintaining a fixed temperature (67°C), and the whole procedure can be finished in 50 min. The LOD of the multiplex MCDA-LFB method was 10 fg per reaction with pure cultures, and 100% specificity was acquired using cultured bacteria. Most importantly, when the multiplex MCDA-LFB was used to detect clinical samples, it showed 88.2% sensitivity and 98.3% specificity.

Choosing an appropriate target is important for the high sensitivity and specificity of the diagnostic assay. IS*6110* and IS*1081* are multi-copy insertion sequences that are specific for MTBC. In the genome of reference strain H37Rv, IS*6110* and IS*1081* copy numbers are 16 and 6, respectively, which makes them useful as diagnostic markers. However, the copy number of IS*6110* is low (even only one copy) or absent in a certain proportion of MTB strains of Southeast Asia (Yuen et al., 1993; Fomukong et al., 1994; Cronin et al., 2001). Another insertion sequence IS*1081* (five to six copies in MTBC strains) was examined as a subsidiary marker (van Soolingen et al., 1992; Yang et al., 1994). The combined detection of IS*1081* can increase the positive rate for such strains. This is the reason that the new generation of Xpert assay, Xpert MTB/RIF ultra, also integrated IS*1081* (Chakravorty et al., 2017; Dorman et al., 2018). In this study, for each target gene, 10 primers were designed that bind to different regions and ensure high specificity. Two sets of primers were labeled in the same way (C1-FITC labeled, D1-biotin labeled). In this case, the amplification products can be detected simultaneously by the anti-FITC on the LFB strip, which not only guarantees the sensitivity but also reduces total cost.

The real-time turbidity analysis and visual agent detection are usually used to indicate the isothermal amplification results. However, the former requires a complex, expensive and precise apparatus, and the later usually produces ambiguous results when the concentration of target sequences is very low (Wang et al., 2018b). In this study, LFB platform was used to analyze the MCDA products. It is rapid (the results can be seen in 2 min), simple (does not need complex equipment) and sensitive (LOD is 10 fg), which is suitable for clinical use and field detection.

To verify the value of multiplex MCDA for clinical application, clinical samples from 179 patients were evaluated and compared with the WHO-recommended method of Xpert MTB/RIF and liquid culture using Bactec MGIT960. According to our assay, the sensitivity was the lowest for liquid culture (42.0%), followed by Xpert MTB/RIF (49.6%) and multiplex MCDA (88.2%) among 119 clinically diagnosed TB patients. We further divided the samples into culture-positive group (50 patients) and culture-negative group (69 patients). Among culture-positive specimens, both Xpert MTB/RIF and multiplex MCDA assay had good sensitivity (86.0% and 98.0%, respectively). The performance of Xpert MTB/RIF showed similar sensitivity with that reported in a recent systematic review (85%) (Horne et al., 2019), indicating that there is no deviation in the selection and detection of specimens. While among the culture-negative samples, the positive rates of Xpert MTB/RIF and multiplex MCDA assay were 23.2% and 81.2%, respectively. Thus, the multiplex MCDA greatly improved the detection rate in culture-negative patients. As a consequence, more TB patients will be early diagnosed using multiplex MCDA assay, and then reduce the transmission possibility.

A concern of this multiplex MCDA assay is the reliability of its positive outcomes, especially for the samples with culture-negative outcomes (Wang et al., 2015). Although we could not clarify those extra positive results of MCDA in this study, the specificity of 98.3% (59/60) could confirm its reliability to some extent. However, since the yield of isothermal amplification is very large, contamination will be a big challenge during application (Wang et al., 2015). Aerosol droplets that contain a high concentration of MCDA products could lead to false positive results in subsequent experiments. Accordingly, special measures should be taken to avoid contamination and false positive outcome.

## Conclusion

A multiplex MCDA-LFB assay targeting both IS*6110* and IS*1081* for MTB detection was successfully developed and validated using pure cultures and clinical samples. It is a rapid and simple diagnostic method with high sensitivity and specificity for *M. tuberculosis* detection. Further prospective study will be needed to evaluate its ability for early TB diagnosis in specific groups, such as children and HIV-infected patients.

## Data Availability

The raw data supporting the conclusions of this manuscript will be made available by the authors, without undue reservation, to any qualified researcher.

## Ethics Statement

The studies involving human participants were reviewed and approved by Ethics committee of the Beijing Children’s Hospital, Capital Medical University and Beijing Chest Hospital, Capital Medical University. Written informed consent to participate in this study was provided by the participants’ legal guardian/next of kin.

## Author Contributions

W-WJ conceived and designed the experiments. W-WJ, YW, Y-CW, JX, LS, J-QL, S-AW, T-TZ, and QM performed the experiments. W-WJ, H-RH, and A-DS contributed to the reagents and materials. W-WJ, H-RH, and A-DS analyzed the data. W-WJ and YW performed the software. W-WJ, YW, H-RH, and A-DS wrote the manuscript. All authors have contributed and approved the final version of the manuscript.

## Conflict of Interest Statement

The authors declare that the research was conducted in the absence of any commercial or financial relationships that could be construed as a potential conflict of interest.
